# Impact of Real-Time Elastography versus Systematic Prostate Biopsy Method on Cancer Detection Rate in Men with a Serum Prostate-Specific Antigen between 2.5 and 10 ng/mL

**DOI:** 10.1155/2013/584672

**Published:** 2013-01-16

**Authors:** Gianluigi Taverna, Paola Magnoni, Guido Giusti, Mauro Seveso, Alessio Benetti, Rodolfo Hurle, Piergiuseppe Colombo, Francesco Minuti, Fabio Grizzi, Pierpaolo Graziotti

**Affiliations:** ^1^Department of Urology, Humanitas Clinical and Research Center, Via Manzoni 56, Rozzano, 20089 Milan, Italy; ^2^Service of Echography, Humanitas Clinical and Research Center, Via Manzoni 56, Rozzano, 20089 Milan, Italy; ^3^Department of Pathology, Humanitas Clinical and Research Center, Via Manzoni 56, Rozzano, 20089 Milan, Italy; ^4^Service of Statistics, Humanitas Clinical and Research Center, Via Manzoni 56, Rozzano, 20089 Milan, Italy; ^5^Laboratory of Molecular Gastroenterology, Humanitas Clinical and Research Center, Via Manzoni 56, Rozzano, 20089 Milan, Italy

## Abstract

The actual gold standard for the diagnosis of prostate cancer includes the serum prostate-specific antigen, the digital rectal examination, and the ultrasound-guided systematic prostate biopsy sampling. In the last years, the real-time elastography has been introduced as an imaging technique to increase the detection rate of prostate cancer and simultaneously reduce the number of biopsies sampled for a single patient. Here, we evaluated a consecutive series of 102 patients with negative digital-rectal examination and transrectal ultrasound, and prostate-specific antigen value ranging between 2.5 ng/mL and 10 ng/mL, in order to assess the impact of real-time elastography versus the systematic biopsy on the detection of prostate cancer. We found that only 1 out of 102 patients resulted true positive for prostate cancer when analysed with real-time elastography. In the other 6 cases, real-time elastography evidenced areas positive for prostate cancer, although additional neoplastic foci were found using systematic biopsy sampling in areas evidenced by real-time elastography as negative. Although additional studies are necessary for evaluating the effectiveness of this imaging technique, the present study indicates that the limited accuracy, sensitivity, and specificity do not justify the routine application of real-time elastography in prostate cancer detection.

## 1. Introduction

Prostate cancer (PC) remains the most common cancer in men in the Western world [1]. The gold standard tools currently applied for the diagnosis of PC include the serum prostate-specific antigen (PSA), the digital rectal examination (DRE), and the ultrasound-guided systematic prostate biopsy sampling [2–4]. Although over the years research has tried to increase the sensitivity and specificity of this multiapproach, PC detection rate is still inadequate [3, 5, 6], being at the first biopsy no more than 20%–30%. New imaging modalities for detecting PC are currently claimed and represent the subject of intensive and continuous research. In the last years, the real-time elastography (RTE) has been introduced as a new technique to increase the detection rate of PC and simultaneously reduce the number of biopsies sampled for single patient [7–10]. Elastography imaging is based on the higher density of cancerous cells and blood vessels, resulting in a major stiffness than that of the natural tissue [11, 12]. Several studies have shown that RTE successfully increases the detection rate of different neoplasia including thyroid, breast, and PC. Salomon et al. reported that positive predictive value (PPV), negative predictive value (NPV), and accuracy for RTE were 87.8%, 59%, and 76%, respectively [6]. They and others, however, claimed the need for further studies and clinical trials to confirm the real impact of RTE in the PC detection [13–17]. Aigner et al. applied RTE in 94 men with PSA value of 1.25 ng/mL or greater and 4.00 ng/mL or less [18] in order to evaluate the peripheral zone tissue elasticity and hard areas defined as suspicious for PC. They found that the cancer detection rate per core was 4.7-fold greater for RTE targeted than systematic biopsy. They concluded that RTE targeted biopsy could be of help to detect PC with a decreased number of biopsy cores compared with that of systematic biopsy [18]. Eggert et al. assess whether elastography-guided prostate biopsies improve the cancer detection in men with suspected prostate cancer, by investigating 351 prospectively randomized patients who underwent prostate biopsies for the first time [19]. They found that the overall cancer detection rate was 39% (137/351): 40.2% (76/189) in the elastography group and 37.7% (61/189) in the control group, respectively. Eggert et al. concluded that elastography did not improve the cancer detection rate in their large cohort of patients [19]. 

Based on these controversial findings, we prospectively evaluated a consecutive series of 102 patients with negative DRE and transrectal ultrasound, and PSA value ranging between 2.5 ng/mL and 10 ng/mL, in order to assess the impact of RTE versus the systematic biopsy on the detection of PC.

## 2. Materials and Methods

### 2.1. Patients

The study involved 102 patients with a mean age of 64.5 years (range: 46–79 years), a median PSA of 5.92 ng/mL (range: 2.5–10 ng/mL), a median prostate volume 45 mL (range: 10–135 mL), and a median free-to-total PSA ratio of 16% (range: 6%–47%).

The Ethics Committee of the Institute enrolling the patients (IRCCS Istituto Clinico Humanitas, Rozzano, Milan, Italy) approved the study, and all patients gave a signed consensus.

Patients who undergo medical treatment with Finasteride or Dutasteride and/or who have experienced symptoms correlated to prostatitis within three months prior to prostate biopsy mapping and/or suffering from urinary tract infections were excluded from the study. Additional exclusion criteria include patients subjected to prostatic adenomectomy (TURP or open adenomectomy).

### 2.2. Real-Time Elastography

**Figure 1:**
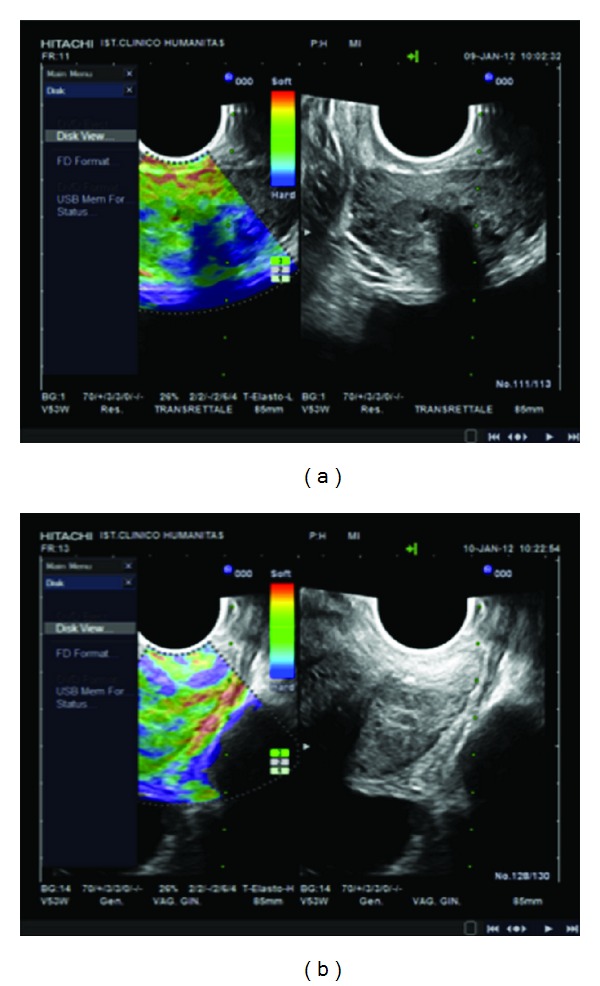
According to a colour gradient, areas of increased tissue stiffness (i.e., blue areas) were considered suspicious for prostate cancer (a), while more elastic areas (i.e., red and green zones) were interpreted as natural prostatic tissue (b).

RTE was done using an ultrasound system (Hitachi EUB 8500, Hitachi Medical, Tokyo, Japan) equipped with an ultrasound trans-rectal probe of 7.5 MHz. The elastogram was provided by a slight compression and decompression of the prostate. According to a colour gradient, areas of increased tissue stiffness (blue areas) were considered suspicious for PC, while more elastic areas (i.e., red and green zones) were interpreted as natural prostatic tissue (Figure 1). All patients had DRE and trans-rectal ultrasound negative for suspicious areas of PC. In case of positive or dubious DRE or trans-rectal ultrasound, the patient was removed from the study. Two experienced sonographers performed a double-blinded RTE to reduce the interpretative subjectivity of the images. Malignancy criteria described by König et al. were adopted to define cancer suspicious areas [20]. In case of agreement between two operators, it was carried out a targeted biopsy of lesions seen throughout the gland and not considering only the peripheral portion. In the case of discrepancy between two operators, the patient was removed from the study, and the biopsy was not undertaken on the areas highlighted with the RTE. Targeted biopsies were performed in all the positive areas recognized by RTE. Furthermore, each patient underwent standard systematic biopsies (13 cores). 

### 2.3. Histopathological Analysis

Biopsy specimens were fixed in 10% formalin, embedded in paraffin, serially cut at 3 *μ*m intervals, and subsequently histochemically stained with a freshly made haematoxylin and eosin solution for the microscopy observation by the same uropathologist (PC). For each patient, anatomoclinical parameters, including the percentage of neoplastic disease, Gleason score, capsular invasion, and surgical margins, were evaluated. 

### 2.4. Statistical Analysis

Sensitivity, specificity, PPV, NPV, and accuracy were estimated for each patient and for each biopsy core (all cores were evaluated). To achieve an accuracy of RTE between 65% and 80% with *α* error of 5% and a power of 90%, according to Fleming's multistep procedure design [21], a minimum enrolment of 75 patients was predicted. All the obtained data were analysed using Stata 9 software (StataCorp LP, USA). 

## 3. Results

**Figure 2:**
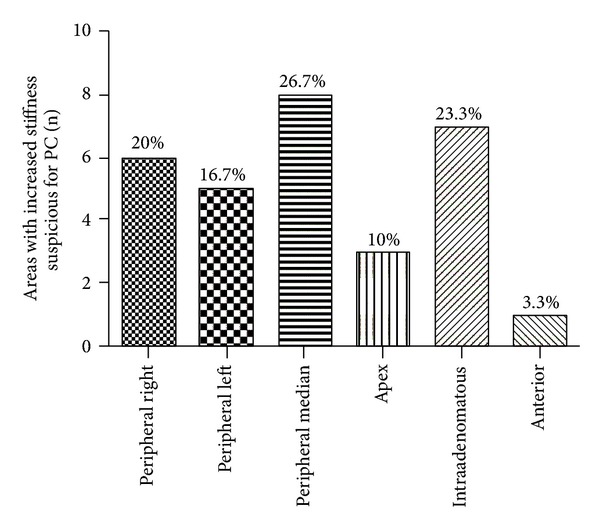
Topographical distribution of areas with increased stiffness suspicious of prostate cancer highlighted by RTE in different anatomical zones of the prostate gland.

A total of 32 out of 102 (31.3%) patients were found positive for PC, 2/102 (1.96%) presented atypical small acinar proliferation (ASAP), and 3/102 (2.94%) had high-grade prostatic intraepithelial neoplasia (HGPIN) at the biopsy. In 30/102 (29.4%) patients, RTE revealed areas of greatest stiffness suspected of PC, specifically located in the prostate zones as follows: apex in 3 patients (10%), intradenomatous in 7 (23.3%) patients, peripheral median in 8 (26.7%) patients, peripheral right in 6 (23.3%) patients, peripheral left in 5 (16.6%) patients, and bilateral device in 1 (3.3%) patient (Figure 2). 

Seven out of 102 patients (6.8%) were found true positives by RTE. Among these, 1 patient (1/102, 0.9%) had PC localized only in an area indicated by RTE. In the other 6 (6/102, 5.8%) patients, PC was detected, not only in areas highlighted by RTE, but also in other sites evidenced by RTE as natural prostatic tissues. Tumoral lesions were subsequently diagnosed by the systematic biopsy method. Histological Gleason grade 3 + 3 was found in 4/7 patients, 3 + 4 in 1/7 patient, 4 + 5 in 1/7 patient, and 5 + 5 in 1/7 patient. True negatives recognized by RTE were 48/102 (47%) patients. Also, 1/48 patients showed foci of ASAP and 1/48 patient foci of HGPIN. 

False positives were 25/102 (24.5%) patients. In 2/25 patients, ASAP was found and in 1/25 HGPIN. False negatives were 22/102 (21.6%) patients, including 1/22 patient showing ASAP, 16/22 with Gleason score 3 + 3, 1/22 patient with Gleason score 3 + 4, 2/22 patients with Gleason score 4 + 3, 1/22 patient with Gleason score 4 + 4, and 1/22 patient with Gleason score 4 + 5. RTE sensitivity, specificity, PPV, and NPV were found to be 24.4%, 65.7%, 21.9%, and 68.6%, respectively. The accuracy was 53.9%.

When considering the single-core biopsy, a total of 1386 cores were histologically analysed. Considering all the patients with suspected areas at RTE, we obtained 60 positive cores of which PC was in 9 (15%) cores. When considering the systematic biopsy cores, we analysed 1326 of which histologically positive for PC were 103 (7.76%) cores.

## 4. Discussion

Here, we have shown that only 1 out of 102 patients resulted true positive for PC when analysed with RTE, that is, only 1 patient had an area really positive for PC in the absence of other neoplastic foci detected by systematic biopsy sampling. In the other 6 cases, RTE evidenced areas positive for PC, although additional neoplastic foci were found using systematic biopsy sampling in areas evidenced by RTE as negative. 

In 22 out of 102 (21.5%) patients, RTE was falsely negative. Although RTE does not recognize suspicious areas for PC, in this subgroup of patients, it was found with systematic biopsy sampling. When we considered the subgroups of “true positives” and “false negatives” patients at RTE, any correlation between Gleason score and RTE was found, that is, high-grade lesions were not preferentially highlighted than those with low Gleason score.

In 25 out of 102 (24.5%) patients, RTE showed “false positive” areas for PC. This result suggests that RTE in about a quarter of cases detects areas that at the subsequent histological analysis resulted without neoplastic foci. These findings and the low RTE accuracy (53.92%) obtained in our study (which was expected between 65% and 80%) seem too low to substitute the actual approach based on the systematic biopsy sampling and limits the routinely application of current RTE technology into clinical practice.

Aigner et al. [18] found that cancer detection rate per core was 4.7-fold greater for targeted than for systematic biopsy. In our study, the sensitivity was 2-fold enhanced. However, we should consider that the total detection rate in our series is 31%, and in 31/32 (96.8%) patients, the diagnosis of PC was made by systematic biopsy and not with RTE. Additionally, although different authors suggested that the higher the grade of the lesion the higher the RTE detection, our findings do not confirm this assumption, because when we analysed the Gleason score, the majority of patients were graded as 3 + 3. The only true positive detected by RTE had a Gleason score of 5 + 5. But if we analyse the high-grade group (Gleason scores: 4 + 3, 4 + 4, and 4 + 5), it appears that only two out of five patients were diagnosed with RTE.

Zhang et al. [22] assessed the diagnostic impact of RTE on differentiating malignant from benign lesions, by analysing a series of 83 patients suspected of having PC in the prostate peripheral zones. Although they showed the significant value of RTE in the diagnosis of PC, additional studies are required to establish the full significance of their findings, as they applied RTE to patients with a PSA value >10 ng/mL and palpable or visible lesions [22]. 

Walz et al. showed that RTE alone did not enable the identification of the PC index lesion with satisfactory reliability [7]. In addition, they stated that RTE had a lower sensibility when compared to the systematic biopsy. Brock et al. [13] concluded that “Further improvement is needed to achieve sensitivity values that can justify the implementation of these techniques within current clinical guideline” and that “The combination of RTE and data from randomized 12 core biopsies allows promising ability to correctly identify the PC index lesion.” Brock et al. evaluated whether RTE-guided biopsy improves PC detection compared to conventional systematic grey scale ultrasound guidance in 353 consecutive patients suspicious for prostate cancer [23]. They concluded that sensitivity to visualize and detect PC improved using RTE in addition to grey scale ultrasound during prostate biopsy, although overall sensitivity did not reach levels to omit a systematic biopsy approach [23]. In a previous study, Brock et al. suggested that RTE enhances grey scale ultrasound, although improvement is still needed to achieve a clinically meaningful sensitivity [13].

The previous studies confirm the actual discrepancies in using RTE to detect PC. As we previously showed in the use of Colour-Doppler ultrasonography with or without contrast agents [24], these controversies might be mainly due to (a) the reproducibility of applied methodologies that are operator-dependent, (b) the variability of the employed equipment, and (c) the criteria used to select the study population. We retain that the subjectivity of the operator in the detection of the prostate zones with higher stiffness is the primary cause for obtaining discrepant results. In this respect, it will be important to reduce the subjective interpretation and standardize as far as possible the employed method [25–27]. Kapoor et al. suggested that combining RTE with trans-rectal ultrasound significantly improves the sensitivity to detect carcinoma prostate in patients with raised PSA; however, RTE is unable to differentiate PC from chronic prostatitis [9]. One of the characteristic findings of elastography is its excellent detection of anterior tumours. The low detection rate of high-grade tumours in this analysis was likely due to the predominance of high-grade tumours in a peripheral location compared to the anterior location of the low-grade tumours [12].

Finally, regarding the variability of the population enrolled in the present study, we tried to eliminate all the conditions that, to the best of our knowledge, might determine incorrect interpretations of PSA levels. It should be underlined that our population was characterized by a serum PSA value ranging from 2.5–10 ng/mL, and were excluded from the study subjects with palpable or visible lesions.

It is indubitable that additional multicentric studies using a higher number of patients are still necessary for evaluating the effectiveness of this imaging technique. To date, however, its routine application in PC detection remains unfounded.

## 5. Conclusions

New imaging modalities for detecting PC are currently claimed and represent the subject of intensive research in the field of the medical sciences. RTE is a well-documented ultrasound modality for detecting the “stiffness,” a physical property of biological tissues. Sensitivity to visualize and detect prostate cancer improved using RTE in addition to grey scale ultrasound during prostate biopsy. Overall sensitivity did not reach levels to omit a systematic biopsy approach. The present study suggests that the limited accuracy, sensitivity, and specificity do not justify the routine application of RTE in PC detection.
